# Important Role of Bacterial Metabolites in Development and Adjuvant Therapy for Hepatocellular Carcinoma

**DOI:** 10.3390/curroncol32120673

**Published:** 2025-11-29

**Authors:** Guixian Ye, Hui Zhang, Qiang Feng, Jianbin Xiao, Jianmin Wang, Jingfeng Liu

**Affiliations:** 1 Innovation Center for Cancer Research, Clinical Oncology School of Fujian Medical University, Fujian Cancer Hospital, Fuzhou 350014, China; ygx200002@163.com (G.Y.);; 2 Fujian Key Laboratory of Advanced Technology for Cancer Screening and Early Diagnosis, Fujian Cancer Hospital, Fuzhou 350014, China; 3 Department of Hepatobiliary and Pancreatic Surgery, Clinical Oncology School of Fujian Medical University, Fujian Cancer Hospital, Fuzhou 350014, China; zh2405@hotmail.com

**Keywords:** hepatocellular carcinoma, gut microbiome, bacterial metabolites, immunometabolism, gut–liver axis, therapeutic strategy

## Abstract

Bacterial metabolites play a dual role in hepatocellular carcinoma (HCC), exhibiting both tumor-promoting and tumor-suppressing activities dictated by their structural diversity. This review synthesizes recent advances in understanding how key microbial metabolites—such as bile acids, short-chain fatty acids, and polyamines—remodel the tumor immune microenvironment through mechanisms including immunometabolic reprogramming, epigenetic modification, and regulation of signaling pathways (e.g., FXR, TLR, and mTOR). We highlight their roles in modulating the function of T cells, NK cells, and tumor-associated macrophages and discuss emerging strategies that target these metabolites—including probiotic interventions, fecal microbiota transplantation, and metabolite-based adjuvants—to enhance immunotherapy efficacy and overcome resistance. By integrating mechanistic insight into translational potential, this work outlines a metabolite–immunometabolism–hepatocarcinogenesis framework and proposes novel combinatorial approaches for HCC treatment.

## Simple Summary

Hepatocellular carcinoma (HCC) remains a highly lethal cancer, with the gut–liver axis playing a pivotal role in its pathogenesis and therapy. This review focuses on bacterial metabolites as key mediators linking the gut microbiota to HCC development, highlighting their dual pro- and anti-tumor effects. Critical metabolites such as bile acids, short-chain fatty acids, lipopolysaccharide, polyamines, and trimethylamine N-oxide (TMAO) regulate HCC progression by remodeling the tumor immune microenvironment, reprogramming immunometabolism, and modulating core signaling pathways including NF-κB, STAT3, and mTOR. We also summarize therapeutic strategies targeting these metabolites—such as probiotic supplementation, fecal microbiota transplantation (FMT), and metabolite modulation—and their synergy with immunotherapy to enhance treatment efficacy. Additionally, we propose a “metabolite–immunometabolism–hepatocarcinogenesis” framework to integrate these mechanisms, offering novel insights to develop personalized HCC prevention and treatment approaches.

## 1. Introduction

The mortality rate caused by hepatocellular carcinoma (HCC) is one of the highest among major cancers, and its five-year survival rate is as low as 20% [1,2]. Patients with chronic liver diseases, such as alcoholic fatty liver disease, non-alcoholic fatty liver disease (NAFLD), type II diabetes mellitus, and viral hepatitis, mainly caused by Hepatitis B virus and Hepatitis C virus, tend to ultimately progress to HCC [3,4]. Malignant drivers, such as liver injury and inflammation, play a non-negligible role in this process [5,6]. The critical role of the bacterial microbiota in promoting the development of liver diseases and the progression of HCC cannot be ignored, and there exists an extremely close bi-directional relationship between the gut microbiota and the liver [7], which is known as the intestinal–microbiota–liver axis, or in short, the gut–liver axis. The profound influence of the gut microbiome on liver cancer pathogenesis, spanning from inflammation to fibrosis and carcinogenesis, has been extensively documented and reviewed [8].

It is critical to distinguish between the roles of the gut microbiota and their derived metabolites within this axis. The gut microbiota exerts its influence primarily through ecological interactions (e.g., competition and colonization resistance) and by modulating the host’s systemic and hepatic immune responses. In contrast, bacterial metabolites—such as bile acids (BAs), short-chain fatty acids (SCFAs), and lipopolysaccharide—act as direct molecular messengers. These small molecules can translocate from the gut, enter the portal circulation, and directly regulate host cell functions in the liver by binding to specific receptors (e.g., FXR, GPCRs, and TLRs) and altering key signaling pathways and epigenetic programs. This dynamic interplay, wherein the microbial community produces metabolites that directly mediate physiological and pathological effects, forms the core mechanistic foundation of the gut–liver axis in HCC pathogenesis [9,10,11].

The intestinal microbiota and its complex metabolites can significantly affect the balance of the liver’s local environment. When the balance of intestinal flora is disrupted, i.e., ecological dysregulation, the risk of various liver diseases increases [9,10]. A study showed that plasma concentrations for certain gut microbiota-derived metabolites were significantly different in HCC patients compared with healthy controls, indicating that these metabolites could be used as potential non-invasive indicators for early detection [12]. An imbalance of the intestinal flora leads to increased intestinal permeability and exacerbates the translocation of bacteria and bacterial ligands, generating an inflammatory environment and inducing liver damage [13,14]. In addition, bacterial metabolites regulate the energy metabolism of host cells and alter signaling pathways to influence the pathological process of HCC, e.g., certain metabolites may affect the processes of cell proliferation, apoptosis, and invasion, which may promote or inhibit the progression of HCC [11].

Patients with chronic viral hepatitis, primarily caused by Hepatitis B virus (HBV) and Hepatitis C virus (HCV), constitute a high-risk population for HCC. Beyond the direct oncogenic effects of the viruses, growing evidence suggests that the gut–liver axis and bacterial metabolites play a crucial role in this process. For instance, gut dysbiosis and increased intestinal permeability are common in chronic hepatitis patients, leading to elevated levels of bacterial products like lipopolysaccharide (LPS) in the portal circulation. This can activate TLR4 signaling in hepatocytes and hepatic stellate cells, fostering a chronic inflammatory and pro-fibrotic microenvironment that accelerates the progression from chronic hepatitis to cirrhosis and ultimately to HCC. Furthermore, alterations in bile acid metabolism by the gut microbiota have been implicated in modulating viral persistence and liver inflammation in HBV/HCV infections, thereby influencing the oncogenic trajectory.

For liver diseases and liver cancer, bacterial metabolites produced by intestinal flora can be classified as pro-cancer or anti-cancer metabolites according to their mode of action and the final clinical results. This study examined the involvement of lipopolysaccharide (LPS), BAs, and other substances in the carcinogenesis of HCC; we also analyzed the inhibitory roles of indoles and polyamines. To achieve a more comprehensive understanding of the influence of intestinal flora and its metabolites, we also discussed the utility and broad application prospects of probiotic flora and their metabolites in supporting the prevention and treatment of HCC.

This review builds upon and extends previous foundational work on the gut–liver axis in HCC [9,10,11] by providing a timely and critical synthesis focused specifically on bacterial metabolites as central mediators. While earlier publications have established the epidemiological and ecological links between the gut microbiome and HCC, our objective is to delve deeper into the molecular mechanisms and therapeutic applications of the metabolites themselves. We place particular emphasis on the burgeoning field of immunometabolism, exploring how metabolites such as BAs, SCFAs, and polyamines directly reprogram the tumor immune microenvironment. Furthermore, this review uniquely bridges these mechanistic insights with the latest translational and clinical advances, including the use of metabolites as adjuvants and the modulation of the gut microbiome to enhance immunotherapy. By framing bacterial metabolites as a dynamic, targetable interface between the host and its microbiota, this review aims to offer a fresh perspective on HCC pathogenesis and open new avenues for combinatorial therapeutic strategies.

## 2. Influence of Bacterial Metabolites on HCC Occurrence and Development

### 2.1. Bacterial Metabolites Promoting Hepatocellular Carcinogenesis and Development

#### 2.1.1. Bile Acids (BAs)

BAs are essential derivatives of cholesterol from hepatic origin. Primary BAs (PBAs) are synthesized in the liver, and secondary BAs (SBAs), such as deoxycholic acid (DCA) and lithocholic acid (LCA), are produced from PBAs by gut microorganisms through dehydroxylation and deconjugation [15,16,17].

**Figure 1:**
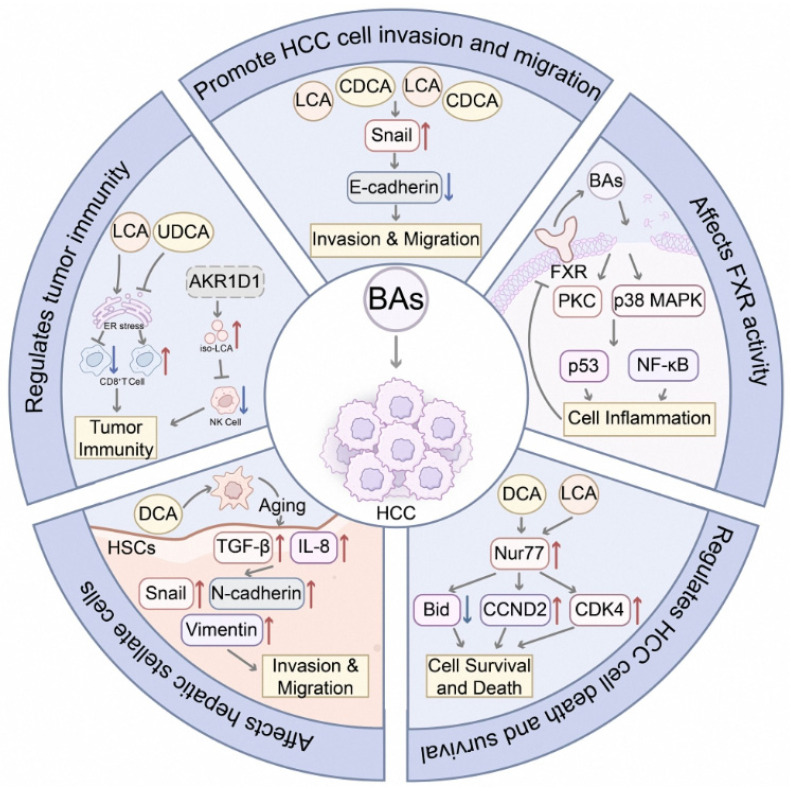
The promoting effects of BAs in HCC. This schematic illustrates the dual and context-dependent functions of bile acids, highlighting their promoting effects through disrupted FXR signaling, induction of EMT, and creation of an immunosuppressive microenvironment, as well as the protective effects mediated by specific bile acids like UDCA. Abbreviations: Aldo-Keto Reductase Family 1 Member D1, AKR1D1; Bile Acid, BA; Chenodeoxycholic Acid, CDCA; Cyclin-Dependent Kinase 4, CDK4; Deoxycholic Acid, DCA; Epithelial–Mesenchymal Transition, EMT; Endoplasmic Reticulum, ER; Farnesoid X Receptor, FXR; Hepatic Stellate Cell, HSC; Interleukin-8, IL-8; Lithocholic Acid, LCA; Natural Killer Cell, NK cell; Primary Bile Acid, PBA; Protein Kinase C, PKC; Secondary Bile Acid, SBA; Senescence-Associated Secretory Phenotype, SASP; Transforming Growth Factor Beta, TGF-β; Ursodeoxycholic Acid, UDCA [18,19,20,21,22,23,24,25,26,27,28].

BAs promote HCC through multiple pathways (Figure 1). The farnesoid X receptor (FXR) is a key cellular receptor for BAs. While normal FXR activity is known to maintain metabolic homeostasis and suppress HCC progression [18,19], its function can be disrupted in diseased livers. For instance, BA-induced inflammation can inhibit FXR signaling, creating a vicious cycle that promotes further BA accumulation and liver damage [20,21]. Specific BAs like CDCA and LCA promote epithelial–mesenchymal transition (EMT) by upregulating Snail and reducing E-cadherin expression [22]. DCA and LCA also induce the expression of nuclear receptor Nur77, regulating cell cycle and apoptosis genes [23]. Furthermore, DCA can trigger a senescent phenotype in hepatic stellate cells, enhancing the secretion of pro-fibrotic and pro-invasive factors [24,25].

BAs have a critical role in the regulation of the tumor immune microenvironment. Recent studies found that the accumulation of certain BAs in HCC impedes tumor-specific T cell responses. While PBAs can induce oxidative stress in T cells, SBAs like LCA inhibit T cell and NK cell function by activating endoplasmic reticulum stress. Notably, ursodeoxycholic acid (UDCA) can reverse this immunosuppressive effect, and the accumulation of iso-LCA, promoted by aldo-keto reductase 1D1 (AKR1D1) deletion, impairs NK cell cytotoxicity [26,27,28]. This dual capacity to modulate immunity underscores the complex role of BAs in HCC.

#### 2.1.2. Short-Chain Fatty Acids (SCFAs)

Besides BAs, SCFAs also influence the progression of HCC. SCFAs are a class of carboxylic acids containing one to six carbon atoms and are produced by intestinal flora through fermentation after degradation of complex carbohydrates to oligosaccharides. As the predominant free anionic form (SCFA) in the colon, SCFAs are almost directly absorbed by intestinal epithelial cells. Acetate, propionate, and butyrate are common, collectively accounting for over 95% of the total intestinal SCFAs, with concentrations ranging from 50 to 200 mmol/L. SCFAs not only provide energy to the intestinal epithelium but also act as regulators of gene expression and participate in signaling through specific receptors, thereby broadly affecting the physiological functions of the host [29,30,31,32].

Patients with non-alcoholic fatty liver disease-associated HCC (NAFLD-HCC) frequently exhibit significant structural abnormalities in their intestinal flora. Metabolomics analysis demonstrated that specific SCFAs produced by the intestinal flora promoted the generation of immunosuppressive regulatory T cells while diminishing the anti-tumor activity of CD8^+^ cytotoxic T cells. This interaction ultimately suppressed the host’s anti-tumor immune response [33,34,35]. Another study found that 3-hydroxybutyrate dehydrogenase 1 enhanced the proliferative capacity of HCC stem cells by catalyzing the modification of histone β-hydroxybutyrylation [36]. Together, these studies provided a novel theoretical framework for understanding the mechanisms underlying HCC development through the lenses of intestinal microecology and epigenetic modification.

#### 2.1.3. Microbial Components and Metabolites Promoting HCC

The promoting effect of gut microbiota on HCC progression is mediated by two major types of bacterial-derived substances: microbial structural components (e.g., cell wall fragments and secretory proteins) that directly activate host signaling pathways and microbial metabolites (e.g., polyamines and trimethylamine-N-oxide) that regulate the hepatic microenvironment. Their distinct mechanisms of action in driving HCC are as follows:

Lipopolysaccharide (LPS), an endotoxin located in the outer membrane of Gram-negative bacteria, is a structural component of bacterial cells and has been documented to increase cancer risk. Seminal work by Dapito et al. [37] established a direct causal link between gut-derived LPS, its receptor TLR4, and HCC promotion in mouse models, providing a foundational mechanism for how bacterial components drive hepatocarcinogenesis. Mechanistically, LPS activates the signal transducer and activator of transcription 3 (STAT3), inducing cells to produce vascular endothelial growth factor and promoting both tumor cell proliferation and angiogenesis in HCC tissues, thereby accelerating malignant progression [38]. Galactose lectin-3 is a cellular target recognized and bound by LPS, leading to activation of the downstream mammalian target of rapamycin complex 1 (mTORC1)-associated signaling pathway; this activates downstream target genes, including glucose transporter protein 1, hexokinase 2, and pyruvate kinase isozyme type M2, promoting glucose uptake and the activation of glycolytic pathways in tumor cells [39]. Additionally, LPS can accelerate the establishment of an inflammatory environment and enhance the proliferation, invasion, and migration of HCC cells. In contrast, ginsenoside Rh4 significantly reduces the effects of LPS and shows promising applications in the treatment of HCC [40]. LPS also increases PD-L1 expression through m6A methylation modification of long non-coding RNA MIR155HG, which ultimately promotes immune escape [41]. Neutrophil Extracellular Traps (NETs) play a crucial role in the immune system’s normal defense mechanisms, as they are rich in histones and antimicrobial proteins that capture and eliminate extracellular pathogens [42]. However, recent findings indicate that NETs formed by neutrophils during LPS-induced activation can be internalized by HCC cells. This internalization further activates the Toll-Like Receptor 4 (TLR4)/Toll-Like Receptor 9 (TLR9)–Cyclooxygenase-2 (COX2) signaling axis, which ultimately contributes to the malignant characteristics of HCC [43].

The pro-tumorigenic effects of bacterial metabolites are particularly significant in the context of chronic viral hepatitis. The compromised intestinal barrier function often observed in HBV/HCV patients facilitates the translocation of microbial products, amplifying their impact on the liver. For example, in models of HCV-related liver disease, LPS not only promotes inflammation through TLR4 but can also synergize with viral proteins to enhance STAT3 activation and oxidative stress, creating a feed-forward loop that drives hepatocarcinogenesis. Similarly, alterations in gut microbiota-derived BAs in chronic hepatitis B patients have been linked to suppressed FXR signaling and impaired hepatoprotection, creating a permissive environment for HCC development. These findings underscore bacterial metabolites as key co-factors in virus-driven HCC, bridging the gap between chronic infection and cancer manifestation.

Recent studies demonstrated that disturbances of polyamine metabolism fostered an immunosuppressive microenvironment and inhibited anti-tumor immune responses, thereby accelerating the progression of HCC [44,45]. Upregulation of spermine synthase in HCC cells was associated with an immunosuppressive microenvironment and predicted poor prognosis. Spermine was shown to induce tumor-associated macrophages to polarize toward the M2 phenotype primarily through the activation of the PI3K-Akt-mTOR-S6K signaling pathway, which subsequently diminished the anti-tumor activity of CD8^+^ T cells. Correspondingly, inhibition of spermine synthesis in combination with immune checkpoint inhibitors offered novel insights for the immunometabolic treatment of HCC [46].

Trimethylamine-n-oxide (TMAO), a metabolite produced by the intestinal flora from phosphatidylcholine-rich foods, is a significant risk factor in the development of malignant tumors, including HCC. TMAO notably enhanced the expression of proteins associated with proliferation, migration, and EMT by activating the MAPK signaling pathway [47,48]. TMAO also activated the ILK/AKT/mTOR signaling pathway, which promoted malignant progression for HCC [49], and the TGF-β/SMAD signaling pathway, inducing the development of EMT synergistically with the presence of the heavy metal cadmium (Cd) [50].

In addition to metabolites, some bacterial proteins were also involved in HCC progression. Recently, it was revealed that *Klebsiella pneumoniae* promoted tumor growth through penicillin-binding protein 1b (PBP1b). After entering the liver through the intestinal barrier, bacterial PBP1b directly bound to the TLR4 receptor on the surface of HCC cells, activating the downstream signaling pathway and tumor growth [51].

### 2.2. Bacterial Metabolites That Inhibit the Development of HCC

#### 2.2.1. BAs with Therapeutic Potential

As introduced in the previous section, the bile acid spectrum exhibits considerable functional diversity, with certain BAs demonstrating potent anti-tumor properties. Ursodeoxycholic acid (UDCA) and its conjugate tauroursodeoxycholic acid (TUDCA) are the most prominent examples.

The anti-HCC mechanisms of UDCA are multifaceted. It inhibits tumor cell proliferation and induces apoptosis by blocking the cell cycle at the G0/G1 phase and modulating the balance of Bcl-2 family proteins, leading to caspase-3 activation [52,53,54]. A recent study developed UDCA-based platinum (IV) conjugates, which triggered severe DNA damage and mitochondria-dependent apoptosis [54]. UDCA also promotes autophagy and can downregulate the immune checkpoint molecule PD-L1, thereby reversing the immunosuppressive milieu and enhancing the infiltration of cytotoxic T cells [55]. Furthermore, UDCA can synergize with conventional therapeutics. The combination of UDCA with sorafenib activated ROS-dependent ERK and promoted STAT3 dephosphorylation [56], while its combination with oxaliplatin diminished the inflammatory response and improved therapeutic efficacy [57].

It is worth noting that BAs can have concentration-dependent effects. For instance, high concentrations of CDCA have been shown to attenuate inflammatory injury in hepatocytes by reducing transaminase activity and inhibiting the expression of IL-6 and TNF-α [58,59,60].

#### 2.2.2. SCFAs

Recently, it was showed that the proportion of Lactobacillus reuteri and the content of SCFAs in the gut microbiota were significantly reduced in an HCC-bearing mouse model, especially the most pronounced acetic acid. The gut microbiota was improved, and acetic acid levels were restored after feces from healthy mice were transplanted. Principally, elevated levels of acetic acid decreased the activity of histone deacetylases, which enhanced the acetylation of sex-determining region Y (SRY)-box transcription factor 13 (Sox13) at lysine residue 13, leading to decreased Sox13 expression and reduced production and secretion of interleukin-17A (IL-17) [61,62]. Furthermore, SCFAs significantly inhibited inflammation through the epidermal growth factor pathway but promoted the expression of tumor suppressor disabled-2 homolog, which slowed down HCC progression [63].

In previous research, the results of a liver cancer intervention strategy showed that echinacea polysaccharide improved gut microbial ratios and promoted a significant increase in the proportion of propionic acid- and butyric-acid-producing intestinal flora (e.g., *E. faecalis* and *Clostridium difficile*). Propionic acid and butyric acid attenuated LPS-mediated inflammatory signaling pathways, i.e., they inhibited the activation of the TLR4/NF-κB axis and decreased the expression of several inflammatory factors, such as IL-6 and migration-associated proteins, including matrix metalloproteinase 2 [64,65].

#### 2.2.3. Other Tumor-Suppressive Metabolites

**Figure 2:**
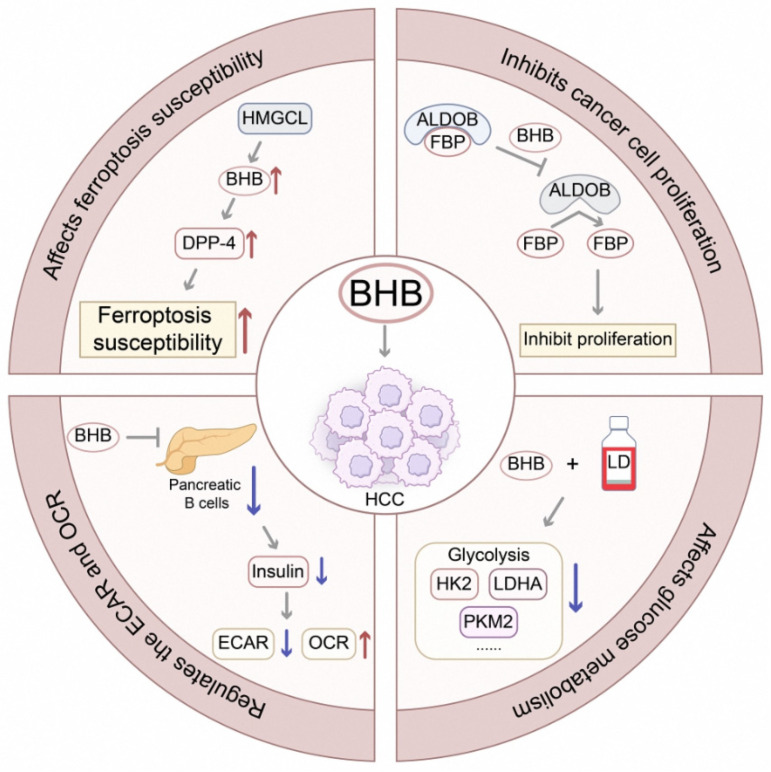
Metabolic pathways for β-hydroxybutyrate (BHB)-mediated suppression of hepatocellular carcinoma. The ketone body BHB, endogenously synthesized by hepatic HMGCL during energy stress, inhibits HCC progression through multiple integrated mechanisms: direct inhibition of the glycolytic enzyme aldolase B (ALDOB); systemic reduction in insulin secretion to shift cellular metabolism from glycolysis (decreased ECAR) to oxidative phosphorylation (increased OCR); and enhancement of DPP4 expression to promote ferroptosis susceptibility. Abbreviations: Aldolase B, ALDOB; β-Hydroxybutyrate, BHB; Dipeptidyl Peptidase-4, DPP4; Extracellular Acidification Rate, ECAR; Fructose-1,6-Bisphosphate, F-1,6-BP; 3-Hydroxymethyl-3-Methylglutaryl-CoA Lyase, HMGCL; Oxygen Consumption Rate, OCR [66,67,68,69].

β-hydroxybutyrate (BHB) is a ketone body endogenously synthesized by the liver under energy stress, such as a low-carbohydrate diet or fasting. Unlike SCFAs, BAs, or other substances directly produced by gut microbiota metabolism, BHB is derived entirely from host hepatic metabolism. However, its role in inhibiting HCC progression by targeting tumor cell metabolism aligns with the core theme of this review—exploring metabolic regulatory mechanisms in the gut–liver axis and identifying potential metabolic targets for HCC therapy—thus providing a valuable complementary perspective on host metabolic interventions for HCC. BHB can inhibit the initiation and progression of HCC through a variety of mechanisms (Figure 2). A previous study found that BHB significantly inhibited aldolase B activity and reduced its ability to bind to the substrate fructose-1,6-bisphosphate, thereby inhibiting the proliferation of cancer cells [66]. Studies [67,68] also revealed that ketogenic diet simulation, i.e., treatment using low-glucose medium combined with BHB, significantly reduced the expression of glycolysis-related proteins in Huh-7 HCC cells. BHB also decreased the extracellular acidification rate by decreasing the secretion of insulin from pancreatic β-cells and increased the rate of cellular oxygen consumption. In contrast, supplementation with exogenous insulin promoted the malignant progression of HCC cells. These results suggested that β-hydroxybutyrate played a key role in inhibiting the proliferation and migration of HCC cells by decreasing insulin production, which provided a new theoretical basis for applying the ketogenic diet in HCC treatment. 3-hydroxymethyl-3-methylglutaryl coenzyme A cleavage enzyme (HMGCL) was shown to play a key catalytic role in the production of β-hydroxybutyrate, increasing the expression level of dipeptidyl peptidase-4 and the Ferroptosis susceptibility of HCC cells [69].

Tryptophan, metabolized by intestinal flora, produces indoles that can influence the progression of HCC. Based on the structure of indoles, derivatives can be designed to inhibit the activity of HCC cells [70]. Indole-2-carboxamide derivatives specifically targeted transient receptor potential canonical-6 and inhibited the growth of HCC cells in a dose-dependent manner, suggesting potential clinical applications [71]. Furthermore, indole-3-carbinol (I3C) exerted anti-cancer effects by inducing apoptosis of HCC cells [72,73]. I3C significantly downregulated miR-21 expression in tumor cells and enhanced the expression of phosphatase and tensin homolog, which inhibited AKT expression [74]. Additionally, I3C could enhance the sensitivity of HCC cells to sorafenib, resulting in improved therapeutic outcomes [75].

Polyamines and their downstream metabolites can serve as potential biomarkers for HCC [76]. Spermidine was shown to prevent liver fibrosis and HCC by activating microtubule-associated protein 1S (MAP1S)-mediated autophagy [77]. N(1), N(11)-diethylnorspermine, an analog of the polyamine spermine, induced polyamine depletion, thereby inhibiting tumor cell growth and the progression of HCC [78]. Clinical data indicated that diacetyl polyamine N1, N12-diacetylspermine could serve as a tumor marker in HCC patients, providing a criterion for assessing disease progression and clinical therapeutic efficacy [79,80]. The novel acylspermine derivative N-(4-aminobutyl)-N-(3-aminopropyl)-8-hydroxy-dodecanamide exhibited a significant inhibitory effect on the malignant behaviors of liver cancer cells [81]. Table 1 presents a summary of these key bacterial metabolites, including their roles and mechanisms.

*Urolithin A* (UA), an intestinal metabolite of ellagic acid, has been shown to significantly reduce the expression of β-catenin and Cyclin D1 in HCC cells. Additionally, UA promoted the expression of the critical tumor suppressor genes p53 and p38 MAPK and the apoptotic markers Cysteine and Aspartate Protein Kinase, which inhibited the proliferation of HCC cells [82,83]. Furthermore, UA could alleviate the symptoms of alcoholic liver disease by modulating the gut–microbe–hepatic axis [84,85]. *Urolithin B* (UB) also exhibited a strong inhibitory effect in HCC progression; it increased the phosphorylation of β-catenin, thereby inhibiting its nuclear–cytoplasmic transport and further suppressing the activity of the Wnt/β-catenin signaling pathway. Additionally, it inhibited the proliferation ability and cell cycle progression of cancer cells while promoting apoptosis [86].

## 3. HCC Therapeutic Strategies Targeting Intestinal Flora and Bacterial Metabolites

**Figure 3:**
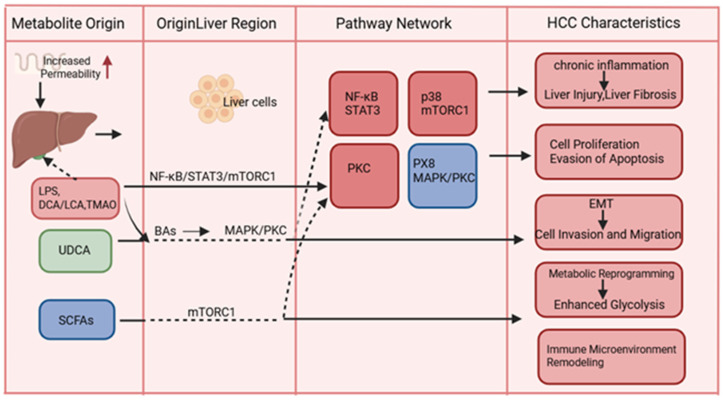
Bacterial metabolites drive hepatocellular carcinoma through core signaling pathway networks. Gut-derived metabolites (LPS, BAs, SCFAs, and TMAO) translocate to the liver via increased intestinal permeability, where they activate or inhibit key pathways (NF-κB, STAT3, mTORC1, MAPK/PKC, and FXR), collectively promoting hallmark HCC features including chronic inflammation, proliferation, EMT, metabolic reprogramming, and immune microenvironment remodeling. Color coding: red (pro-tumor effects: LPS, DCA, LCA, and TMAO); green (anti-tumor effects: UDCA and butyrate); blue (dual roles: SCFAs and FXR). Solid arrows: direct activation; dashed arrows: indirect regulation. Abbreviations: BAs, bile acids; DCA, deoxycholic acid; EMT, epithelial–mesenchymal transition; FXR, farnesoid X receptor; HCC, hepatocellular carcinoma; LCA, lithocholic acid; LPS, lipopolysaccharide; SCFAs, short-chain fatty acids; TMAO, trimethylamine N-oxide; UDCA, ursodeoxycholic acid.

The profound connection between the intestinal flora and its metabolites opens up avenues for the development of diagnostic, therapeutic, and interventional measures for chronic liver disease and HCC. Numerous studies have established that novel strategies targeting the gut microbiota and bacterial metabolites, in conjunction with current HCC therapies, can inhibit the malignant progression of HCC and improve patient prognosis and quality of life (Figure 3).

### 3.1. Therapeutic Strategies Targeting Gut Microbiota

NAFLD-HCC represents a significant category of liver cancers that poses a serious threat to patient health. Supplementation with bifidobacterium pseudolongum suppressed the malignant progression of NAFLD-HCC through the production and secretion of acetate via the gut–hepatic axis [87,88]. Valeric acid produced by Lactobacillus acidophilus rebuilt the intestinal barrier and exerted a preventive effect against NAFLD-HCC by inhibiting the Rho-GTPase signaling pathway [89]. Good liver function is crucial for the recovery of patients with HCC. Oral administration of Bifidobacterium longum has been shown to significantly reduce the severity of liver inflammation and fibrosis while promoting the proliferation and regeneration of normal liver cells, ultimately improving the prognosis for HCC patients [90,91]. Lactobacillus brevis regulated Matrix Metalloproteinase 9 and NOTCH1-related signaling pathways, which ultimately prevented the malignant progression of HCC through production of BAs [92]. Evidence supporting microbiome-targeting interventions demonstrates a clear translational pathway from preclinical models to human trials. While most mechanistic insights (e.g., acetate-mediated suppression of NAFLD-HCC and valeric acid’s barrier protection) originate from animal studies, their clinical relevance is increasingly supported by human trials. The postoperative recovery of liver function with Bifidobacterium longum in HCC patients provides direct clinical validation, while ongoing trials with defined probiotic strains (NCT05378230) represent a critical next step in translating preclinical findings into standardized clinical applications.

Fecal microbiota transplantation (FMT) by transferring fecal material from a healthy donor to a recipient is able to restore a relatively balanced intestinal flora—its core value in HCC therapy lies in optimizing immunotherapy efficacy and reversing immune checkpoint inhibitor (ICI) resistance [93]. In animal models bearing tumors, FMT using microbiota from donors who responded positively to ICI treatment was shown to boost the anti-tumor activity of anti-PD-(L)1 agents and strengthen T cell-driven anti-tumor responses. In contrast, FMT with microbiota from donors unresponsive to ICIs did not yield such favorable outcomes [94,95,96].

### 3.2. Therapeutic Strategies Targeting Bacterial Metabolites

The translational potential of targeting bacterial metabolites is increasingly supported by clinical evidence, underscoring their promise as novel therapeutic agents or adjuvants. Several clinical trials are exploring the modulation of the gut microbiome and its metabolic output to improve outcomes in HCC patients. For instance, fecal microbiota transplantation (FMT) from responsive donors is being investigated in combination with immune checkpoint inhibitors (e.g., anti-PD-1 therapy) to reverse immunotherapy resistance in advanced HCC (NCT05750030). Similarly, probiotic interventions, such as the administration of specific Lactobacillus plantarum strains (NCT05378230), aim to reconstitute a healthy gut ecosystem and promote the production of beneficial metabolites like SCFAs. These clinical efforts directly link the manipulation of bacterial metabolites to tangible therapeutic benefits, paving the way for their integration into standard HCC treatment paradigms.

Bacterial metabolites are key mediators of the gut–liver axis, with dual pro- and anti-cancer roles in HCC. Targeting the synthesis, metabolism, or signaling pathways of these metabolites enables direct regulation of HCC progression.

#### 3.2.1. Modulation of BA Metabolism

Based on the important roles of BAs in the occurrence and development of HCC, regulation of BA metabolism may present new strategies for the treatment of HCC. Recent findings have shown that by activating BA-related receptors such as FXR, the synthesis and secretion of BAs can be effectively reduced, and accumulation of BAs in the liver can be reduced, thus minimizing hepatocellular injury and inflammatory response and inhibiting the progression of HCC [97,98]. The transition from mechanistic understanding to therapeutic application is particularly advanced for BA-targeting approaches. Preclinical models have elucidated how FXR activation inhibits HCC progression and how BA depletion enhances anti-tumor immunity. These findings are now being translated clinically, with BA levels emerging as potential biomarkers for predicting response to combination therapies (e.g., TKIs with anti-PD-1), demonstrating how mechanistic insights from animal studies can directly inform clinical decision-making. As previously described, BAs can influence the immune microenvironment of HCC by regulating immune cells to inhibit the progression of HCC. Inhibition of BA synthesis enhanced the activity of NK cells and Cytotoxic T Lymphocytes (CTLs), thereby inhibiting the growth of HCC [26]. In addition, BAs can be a biomarker for HCC response to targeted therapies. One study found that peripheral blood BA levels correlated with HCC patients’ response to tyrosine kinase inhibitors in combination with PD-1 therapy, and changes in BA levels could serve as a potential biomarker for predicting the effectiveness of treatment [99]. Regulating the expression of genes related to BA metabolism using gene editing technology also provided new ideas for the treatment of HCC [100].

#### 3.2.2. Regulation of SCFAs

SCFAs are major anti-cancer metabolites produced by intestinal flora fermentation, and their therapeutic potential in HCC is mainly reflected in “exogenous metabolite supplementation” and “therapeutic prebiotic-mediated SCFA enhancement”.

Based on the mechanism that acetic acid inhibits HDAC and reduces IL-17 (Section 2.2.2), exogenous acetic acid administration synergizes with immune checkpoint inhibitors. Preclinical studies confirm that this combination not only reduces the pro-inflammatory TME but also enhances the infiltration of CD8^+^ T cells, achieving more significant tumor suppression than monotherapy [61]. Echinacea polysaccharide, as a therapeutic prebiotic, promotes the proliferation of propionic acid- and butyric-acid-producing flora to increase intestinal SCFA levels (Section 2.2.2). In HCC mouse models, this intervention reduces liver tumor burden by inhibiting the TLR4/NF-κB axis, consistent with its ability to dampen inflammatory signaling and restrict tumor cell migration [64,65].

Notably, BAs and SCFAs exhibit dose-dependent effects. Thus, personalized therapeutic dosage adjustment tailored to patients’ gut microbial profiles is crucial for both BA-based and SCFA-based therapies [15,101].

### 3.3. Combined Therapeutic Strategies

The concept of adjuvant therapy in HCC, aimed at preventing recurrence after radical interventions like resection or ablation, is a critical area of investigation. The landmark STORM trial, a phase III study, evaluated the tyrosine kinase inhibitor sorafenib as adjuvant therapy for patients with a high risk of recurrence following resection or local ablation. Although the trial did not meet its primary endpoint of improving recurrence-free survival, it established a benchmark for future adjuvant studies and highlighted the challenges in this setting, including patient selection and the need for more effective strategies [102]. In the evolving landscape, the exploration of novel adjuvants has expanded to include immunomodulatory approaches. Building on this foundation, contemporary research is now focused on how interventions targeting the gut microbiota and bacterial metabolites—by reshaping the tumor immune microenvironment—could potentially fulfill this unmet need as adjuvants to prevent post-operative recurrence or synergize with established therapies.

The synergy between gut microbiota/metabolite interventions and conventional therapies (e.g., immunotherapy) has become a key direction for optimizing HCC treatment. Immunotherapy has opened new horizons and established new therapeutic benchmarks in the treatment of HCC. Notably, atilizumab and bevacizumab represent landmark advancements in HCC immunotherapy [103,104]. Several studies have demonstrated that the complexity of the tumor microenvironment (TME) of HCC is significantly influenced by gut microbes, which in turn affects patient responses to immunotherapy [105,106,107]. Importantly, a significant difference in gut microbiota composition was characterized between HCC patients who responded to immunotherapy and those who did not [108]. This underscored the potential role of gut microbiota as a clinical prognostic marker or therapeutic target for HCC regarding immunotherapy [109,110]. A clinical phase II trial (NCT05750030) is currently underway to evaluate the efficacy and safety of FMT in combination with atilizumab and bevacizumab in patients with advanced HCC.

A typical example of such synergy is the combination of Akkermansia muciniphila (Akk) with anti-PD-1 therapy. Studies have demonstrated that supplementation with Akk reduced the accumulation of immunosuppressive cells, such as monocytic myeloid-derived suppressor cells and M2-type macrophages. In a MAFLD-HCC mouse model, the combination treatment using Akk and PD-1 enhanced T-cell infiltration and activation, resulting in more pronounced anti-tumor effects. The integration of gut microbiome modulation with conventional therapies highlights both the promise and challenges of translational research. Preclinical models have convincingly demonstrated that Akk supplementation enhances anti-PD-1 efficacy, while clinical observations confirm that low Akkermansia abundance correlates with poor immunotherapy response in patients. This parallel evidence from bench to bedside strongly supports the potential of microbiota-based biomarkers and adjuvants, though larger prospective trials are needed to establish causal relationships in human populations. Clinical studies indicated that low levels of Akk were associated with resistance to PD-1 treatment and poor progression-free survival. Akk was not only implicated in the immune escape of MAFLD-HCC but was also anticipated to serve as a potential biomarker for predicting responses to PD-1 immunotherapy [111,112,113].

Importantly, the application of strategies targeting bacterial metabolites holds significant promise in the specific clinical scenario of post-radical therapy (e.g., following curative resection or ablation). The primary goal in this setting is to prevent disease recurrence by eradicating minimal residual disease and modulating the pro-tumorigenic microenvironment. Here, interventions such as probiotic supplementation, FMT, or direct metabolite administration could be deployed as novel adjuvant therapies. By rapidly restoring a healthy gut–liver axis and promoting the production of anti-cancer metabolites (e.g., SCFAs and UDCA), these approaches aim to re-establish systemic immune homeostasis, eliminate lingering immunosuppressive signals, and create a hostile soil for residual cancer cells, thereby potentially improving recurrence-free survival. This represents a paradigm shift from directly killing tumor cells to proactively shaping the host’s internal environment to resist cancer recurrence, addressing a critical unmet need in the current HCC treatment landscape. These diverse adjuvant strategies targeting the gut microbiota and bacterial metabolites are summarized in Table 2.

Despite the promising synergy, the translational application of these combined strategies faces challenges, including substantial inter-individual variability in gut microbiota composition, the influence of host genetics, and the modulating effects of dietary habits, all of which can impact treatment efficacy and reproducibility.

## 4. Summary and Outlook

Drawing upon the evidence discussed in this review, we propose a “metabolite–immunometabolism–hepatocarcinogenesis (MIH) framework” to conceptually integrate the core mechanisms. This framework posits that gut bacterial metabolites are not merely byproducts but central regulators that orchestrate hepatocarcinogenesis by directly reprogramming host immunometabolism. Specifically, metabolites (e.g., BAs, SCFAs, and polyamines) can dictate the fate and function of immune cells within the liver by altering their metabolic pathways (e.g., glycolysis and oxidative phosphorylation), thereby shaping an immunosuppressive or anti-tumor microenvironment. This metabolite-driven immunometabolic reprogramming forms a critical bridge connecting gut microbial ecology to hepatic oncogenesis, providing a unified perspective for understanding how the gut–liver axis influences HCC development and developing targeted interventions.

The “gut–liver axis”, an important physiological regulatory system, connects the intestinal microbiota and its metabolites with the liver through a complex, dynamic, and closely interacting network. This axis is crucial for maintaining normal liver physiological functions, regulating the progression of chronic liver diseases, and facilitating the treatment of HCC. Relevant interventions include targeted regulation of intestinal flora, modulation of specific microbial metabolites, development of inhibitors or agonists for key regulatory molecules in the signaling pathway, and the application of probiotics, prebiotics, and dietary optimization to regulate intestinal microecology and enhance liver function. Additionally, FMT, which aims to reshape intestinal flora, enhance liver homeostasis, and regulate the immune microenvironment, has demonstrated clinical potential. However, its long-term safety and stable efficacy require more systematic and in-depth study.

In summary, the enterohepatic axis system, as a multilevel and multidimensional regulatory network, plays a vital role in maintaining liver health and intervening with liver diseases. Future studies should aim to develop novel and feasible diagnostic and therapeutic strategies to improve the prognosis of patients with liver disease and HCC.

## Figures and Tables

**Table 1:** Summary of Key Metabolites in HCC Pathogenesis and Therapy.

Metabolite Class	Examples	Origin	Role in HCC	Key Mechanisms	References
Bile Acids	DCA, LCA, CDCA, UDCA	Host synthesis + gut microbial modification	Dual role (Pro/Anti-tumor)	- Activate FXR, p38 MAPK, PKC - Induce oxidative/ER stress in immune cells - Promote EMT via Snail - UDCA: induces apoptosis, inhibits HDAC	[15,16,17,18,19,20,21,22,23,24,25,26,27,28,52,53,54,55,56,57]
Short-Chain Fatty Acids	Acetate, Butyrate, Propionate	Gut microbial fermentation of fiber	Dual role (Context-dependent)	- HDAC inhibition - GPCR signaling - Promote Treg differentiation (pro-tumor) - Inhibit IL-17 production (anti-tumor)	[29,30,31,32,33,34,35,61,62,63,64,65]
Lipopolysaccharide	LPS	Gram-negative bacterial membrane	Pro-tumor	- TLR4 activation - STAT3/NF-κB signaling - Promotes angiogenesis, EMT, immune escape	[38,39,40,41,42,43]
Polyamines	Spermine, Spermidine	Gut microbial synthesis + host	Dual role (Pro/Anti-tumor)	- Promote M2 macrophage polarization - Inhibit CD8^+^ T cell function - Spermidine: induces autophagy (protective)	[44,45,46,76,77,78,79,80,81]
Other Metabolites	Indole-3-carbinol, Urolithins	Gut microbial metabolism	Anti-tumor	- Induce apoptosis - Inhibit Wnt/β-catenin signaling - Enhance sorafenib sensitivity	[70,71,72,73,74,75,82,83,84,85,86]
	TMAO, β-hydroxybutyrate	Dietary precursor metabolism, host liver	Pro-tumor (TMAO) Anti-tumor (BHB)	- TMAO: activates MAPK, ILK/AKT/mTOR - BHB: inhibits glycolysis, induces ferroptosis	[47,48,49,50,66,67,68,69]

Bile Acid, BA; Chenodeoxycholic Acid, CDCA; Deoxycholic Acid, DCA; Epithelial–Mesenchymal Transition, EMT; Endoplasmic Reticulum, ER; Farnesoid X Receptor, FXR; G Protein-Coupled Receptor, GPCR; Histone Deacetylase, HDAC; Hepatocellular Carcinoma, HCC; Interleukin, IL; Lithocholic Acid, LCA; Mitogen-Activated Protein Kinase, MAPK; Nuclear Factor Kappa-Light-Chain-Enhancer of Activated B Cells, NF-κB; Protein Kinase C, PKC; Signal Transducer and Activator of Transcription 3, STAT3; Toll-Like Receptor 4, TLR4; Trimethylamine N-Oxide, TMAO; Regulatory T Cell, Treg; Ursodeoxycholic Acid, UDCA.

**Table 2:** Summary of adjuvant interventions targeting gut microbiota and bacterial metabolites in HCC management.

Intervention Category	Specific Strategy/Agent	Mechanism of Action	Stage of Evidence
Probiotics	*Bifidobacterium pseudolongum*	Produces acetate via the gut-liver axis to suppress NAFLD-HCC [87,88].	Preclinical/Animal studies
	*Lactobacillus acidophilus*	Produces valeric acid, rebuilds intestinal barrier, inhibits Rho-GTPase signaling [89].	Preclinical/Animal studies
	*Bifidobacterium longum*	Reduces liver inflammation and fibrosis, promotes hepatocyte regeneration [90,91].	Clinical study in postoperative patients
	*Lactobacillus brevis*	Modulates BAs and regulates MMP-9/NOTCH1 pathways [92].	Preclinical/Animal studies
	Mixed *Lactobacillus plantarum* strains	Aims to improve gut microecology and metabolic output (NCT05378230).	Clinical trial
Fecal Microbiota Transplantation (FMT)	FMT from ICI-responsive donors	Reshapes gut microbiota to reverse ICI resistance and enhance anti-tumor immunity [93,94,95,96].	Preclinical/Clinical trial (NCT05750030)
Bacterial Metabolite-Targeting	Modulation of Bile Acids (e.g., FXR agonists)	Reduces BA synthesis and accumulation, alleviates inflammation, and enhances NK/CD8^+^ T cell activity [26,97,98].	Preclinical/Translational
	Supplementation/Induction of SCFAs (e.g., Acetate, Butyrate)	Inhibits HDAC, reduces IL-17 production, and synergizes with ICIs [61,64].	Preclinical
	Prebiotics (e.g., Echinacea polysaccharide)	Promotes SCFA-producing flora, inhibits TLR4/NF-κB axis [64,65].	Preclinical/Animal studies
Combination Therapy	Akkermansia muciniphila (Akk) + anti-PD-1	Reduces immunosuppressive cells, enhances T-cell infiltration and activation [111,112,113].	Preclinical/Translational

Bile Acids, BAs; Farnesoid X Receptor, FXR; Histone Deacetylase, HDAC; Hepatocellular Carcinoma, HCC; Immune Checkpoint Inhibitor, ICI; Interleukin-17, IL-17; Non-Alcoholic Fatty Liver Disease-Associated Hepatocellular Carcinoma, NAFLD-HCC; Nuclear Factor Kappa-Light-Chain-Enhancer Of Activated B Cells, NF-κB; Natural Killer Cell, NK cell; Short-Chain Fatty Acids, SCFAs; Toll-Like Receptor 4, TLR4.

## Data Availability

No new data were created or analyzed in this study.

## References

[1] Cranford H.M., Jones P.D., Wong R.J., Liu Q., Kobetz E.N., Reis I.M., Koru-Sengul T., Pinheiro P.S. (2024). Hepatocellular Carcinoma Etiology Drives Survival Outcomes: A Population-Based Analysis. Cancer Epidemiol. Biomarkers Prev..

[2] Devarbhavi H., Asrani S.K., Arab J.P., Nartey Y.A., Pose E., Kamath P.S. (2023). Global Burden of Liver Disease: 2023 Update. J. Hepatol..

[3] Mozaffari S., Aliari M., Emamgholipour S., Hosseini H., Amirkiasar P.R., Zare M., Katsiki N., Panahi G., Sahebkar A. (2024). The Effect of Probiotic Consumption on Lipid Profile, Glycemic Index, Inflammatory Markers, and Liver Function in Nafld Patients: A Systematic Review and Meta-Analysis of Randomized Controlled Trials. J. Diabetes Complicat..

[4] Brar G., Greten T.F., Graubard B.I., McNeel T.S., Petrick J.L., McGlynn K.A., Altekruse S.F. (2020). Hepatocellular Carcinoma Survival by Etiology: A Seer-Medicare Database Analysis. Hepatol. Commun..

[5] Yang S., Qiu X., Yang Y., Wu J., Wang S., Zheng B., Wu J., Zhou T., Zhang Y., Bai M. (2025). Lta4h Improves the Tumor Microenvironment and Prevents Hcc Progression Via Targeting the Hnrnpa1/Ltbp1/Tgf-Β Axis. Cell Rep. Med..

[6] Jo Y.M., Son Y.J., Kim S.A., Lee G.M., Ahn C.W., Park H.O., Yun J.H. (2024). Lactobacillus Gasseri Bnr17 and Limosilactobacillus Fermentum Abf21069 Ameliorate High Sucrose-Induced Obesity and Fatty Liver Via Exopolysaccharide Production and Β-Oxidation. J. Microbiol..

[7] Sun D., Xie C., Zhao Y., Liao J., Li S., Zhang Y., Wang D., Hua K., Gu Y., Du J. (2024). The Gut Microbiota-Bile Acid Axis in Cholestatic Liver Disease. Mol. Med..

[8] Schwabe R.F., Greten T.F. (2020). Gut Microbiome in Hcc—Mechanisms, Diagnosis and Therapy. J. Hepatol..

[9] Shen S., Khatiwada S., Behary J., Kim R., Zekry A. (2022). Modulation of the Gut Microbiome to Improve Clinical Outcomes in Hepatocellular Carcinoma. Cancers.

[10] Marroncini G., Naldi L., Martinelli S., Amedei A. (2024). Gut-Liver-Pancreas Axis Crosstalk in Health and Disease: From the Role of Microbial Metabolites to Innovative Microbiota Manipulating Strategies. Biomedicines.

[11] Wu N., Bayatpour S., Hylemon P.B., Aseem S.O., Brindley P.J., Zhou H. (2025). Gut Microbiome and Bile Acid Interactions: Mechanistic Implications for Cholangiocarcinoma Development, Immune Resistance, and Therapy. Am. J. Pathol..

[12] Banerjee R., Wehrle C.J., Wang Z., Wilcox J.D., Uppin V., Varadharajan V., Mrdjen M., Hershberger C., Reizes O., Yu J.S. (2024). Circulating Gut Microbe-Derived Metabolites Are Associated with Hepatocellular Carcinoma. Biomedicines.

[13] Behary J., Raposo A.E., Amorim N.M.L., Zheng H., Gong L., McGovern E., Chen J., Liu K., Beretov J., Theocharous C. (2021). Defining the Temporal Evolution of Gut Dysbiosis and Inflammatory Responses Leading to Hepatocellular Carcinoma in Mdr2 -/- Mouse Model. BMC Microbiol..

[14] Dong L., Luo P., Zhang A. (2024). Intestinal Microbiota Dysbiosis Contributes to the Liver Damage in Subchronic Arsenic-Exposed Mice. Acta Biochim. Biophys. Sin..

[15] Režen T., Rozman D., Kovács T., Kovács P., Sipos A., Bai P., Mikó E. (2022). The Role of Bile Acids in Carcinogenesis. Cell Mol. Life Sci..

[16] Kouhzad M., Götz F., Navidifar T., Taki E., Ghamari M., Mohammadzadeh R., Seyedolmohadesin M., Bostanghadiri N. (2025). Carcinogenic and Anticancer Activities of Microbiota-Derived Secondary Bile Acids. Front. Oncol..

[17] Fleishman J.S., Kumar S. (2024). Bile Acid Metabolism and Signaling in Health and Disease: Molecular Mechanisms and Therapeutic Targets. Signal Transduct. Target. Ther..

[18] Tomioka I., Ota C., Tanahashi Y., Ikegami K., Ishihara A., Kohri N., Fujii H., Morohaku K. (2024). Loss of the DNA-Binding Domain of the Farnesoid X Receptor Gene Causes Severe Liver and Kidney Injuries. Biochem. Biophys. Res. Commun..

[19] Wolfe A., Thomas A., Edwards G., Jaseja R., Guo G.L., Apte U. (2011). Increased Activation of the Wnt/Β-Catenin Pathway in Spontaneous Hepatocellular Carcinoma Observed in Farnesoid X Receptor Knockout Mice. J. Pharmacol. Exp. Ther..

[20] Anwer M.S. (2012). Intracellular Signaling by Bile Acids. J. Biosci..

[21] Gadaleta R.M., Oldenburg B., Willemsen E.C., Spit M., Murzilli S., Salvatore L., Klomp L.W., Siersema P.D., van Erpecum K.J., van Mil S.W. (2011). Activation of Bile Salt Nuclear Receptor Fxr Is Repressed by Pro-Inflammatory Cytokines Activating Nf-Κb Signaling in the Intestine. Biochim. Biophys. Acta.

[22] Fukase K., Ohtsuka H., Onogawa T., Oshio H., Ii T., Mutoh M., Katayose Y., Rikiyama T., Oikawa M., Motoi F. (2008). Bile Acids Repress E-Cadherin through the Induction of Snail and Increase Cancer Invasiveness in Human Hepatobiliary Carcinoma. Cancer Sci..

[23] Hu Y., Chau T., Liu H.X., Liao D., Keane R., Nie Y., Yang H., Wan Y.J. (2015). Bile Acids Regulate Nuclear Receptor (Nur77) Expression and Intracellular Location to Control Proliferation and Apoptosis. Mol. Cancer Res..

[24] Nguyen P.T., Kanno K., Pham Q.T., Kikuchi Y., Kakimoto M., Kobayashi T., Otani Y., Kishikawa N., Miyauchi M., Arihiro K. (2020). Senescent Hepatic Stellate Cells Caused by Deoxycholic Acid Modulates Malignant Behavior of Hepatocellular Carcinoma. J. Cancer Res. Clin. Oncol..

[25] Yoshimoto S., Loo T.M., Atarashi K., Kanda H., Sato S., Oyadomari S., Iwakura Y., Oshima K., Morita H., Hattori M. (2013). Obesity-Induced Gut Microbial Metabolite Promotes Liver Cancer through Senescence Secretome. Nature.

[26] Varanasi S.K., Chen D., Liu Y., Johnson M.A., Miller C.M., Ganguly S., Lande K., LaPorta M.A., Hoffmann F.A., Mann T.H. (2025). Bile Acid Synthesis Impedes Tumor-Specific T Cell Responses During Liver Cancer. Science.

[27] Wei H., Suo C., Gu X., Shen S., Lin K., Zhu C., Yan K., Bian Z., Chen L., Zhang T. (2025). Akr1d1 Suppresses Liver Cancer Progression by Promoting Bile Acid Metabolism-Mediated Nk Cell Cytotoxicity. Cell Metab..

[28] Zheng D., Zhang H., Zheng X., Zhao A., Jia W. (2024). Novel Microbial Modifications of Bile Acids and Their Functional Implications. Imeta.

[29] Martindale R.G., Mundi M.S., Hurt R.T., McClave S.A. (2025). Short-Chain Fatty Acids in Clinical Practice: Where Are We?. Curr. Opin. Clin. Nutr. Metab. Care.

[30] Koh A., De Vadder F., Kovatcheva-Datchary P., Bäckhed F. (2016). From Dietary Fiber to Host Physiology: Short-Chain Fatty Acids as Key Bacterial Metabolites. Cell.

[31] Kimura I., Ichimura A., Ohue-Kitano R., Igarashi M. (2020). Free Fatty Acid Receptors in Health and Disease. Physiol. Rev..

[32] Lu S., Wang C., Ma J., Wang Y. (2024). Metabolic Mediators: Microbial-Derived Metabolites as Key Regulators of Anti-Tumor Immunity, Immunotherapy, and Chemotherapy. Front. Immunol..

[33] Behary J., Amorim N., Jiang X.T., Raposo A., Gong L., McGovern E., Ibrahim R., Chu F., Stephens C., Jebeili H. (2021). Gut Microbiota Impact on the Peripheral Immune Response in Non-Alcoholic Fatty Liver Disease Related Hepatocellular Carcinoma. Nat. Commun..

[34] Yin Q., Ni J.J., Ying J.E. (2024). Potential Mechanisms and Targeting Strategies of the Gut Microbiota in Antitumor Immunity and Immunotherapy. Immun. Inflamm. Dis..

[35] Gao X., Jiang J. (2024). Exploring the Regulatory Mechanism of Intestinal Flora Based on Pd-1 Receptor/Ligand Targeted Cancer Immunotherapy. Front. Immunol..

[36] Zhang H., Chang Z., Qin L.N., Liang B., Han J.X., Qiao K.L., Yang C., Liu Y.R., Zhou H.G., Sun T. (2021). Mta2 Triggered R-Loop Trans-Regulates Bdh1-Mediated Β-Hydroxybutyrylation and Potentiates Propagation of Hepatocellular Carcinoma Stem Cells. Signal Transduct. Target. Ther..

[37] Dapito D.H., Mencin A., Gwak G.Y., Pradere J.P., Jang M.K., Mederacke I., Caviglia J.M., Khiabanian H., Adeyemi A., Bataller R. (2012). Promotion of Hepatocellular Carcinoma by the Intestinal Microbiota and Tlr4. Cancer Cell.

[38] Wang Z., Yan M., Li J., Long J., Li Y. (2019). Zhang. Dual Functions of Stat3 in Lps-Induced Angiogenesis of Hepatocellular Carcinoma. Biochim. Biophys. Acta Mol. Cell Res..

[39] Chen X., Yu C., Liu X., Liu B., Wu X., Wu J., Yan D., Han L., Tang Z., Yuan X. (2022). Intracellular Galectin-3 Is a Lipopolysaccharide Sensor That Promotes Glycolysis through Mtorc1 Activation. Nat. Commun..

[40] Jiang R., Luo S., Zhang M., Wang W., Zhuo S., Wu Y., Qiu Q., Yuan Y., Jiang X. (2023). Ginsenoside Rh4 Inhibits Inflammation-Related Hepatocellular Carcinoma Progression by Targeting Hdac4/Il-6/Stat3 Signaling. Mol. Genet. Genom..

[41] Peng L., Pan B., Zhang X., Wang Z., Qiu J., Wang X., Tang N. (2022). Lipopolysaccharide Facilitates Immune Escape of Hepatocellular Carcinoma Cells Via M6a Modification of Lncrna mir155hg to Upregulate Pd-L1 Expression. Cell Biol. Toxicol..

[42] Zhu W., Fan C., Dong S., Li X., Chen H., Zhou W. (2023). Neutrophil Extracellular Traps Regulating Tumorimmunity in Hepatocellular Carcinoma. Front. Immunol..

[43] Yang L.Y., Luo Q., Lu L., Zhu W.W., Sun H.T., Wei R., Lin Z.F., Wang X.Y., Wang C.Q., Lu M. (2020). Increased Neutrophil Extracellular Traps Promote Metastasis Potential of Hepatocellular Carcinoma Via Provoking Tumorous Inflammatory Response. J. Hematol. Oncol..

[44] Liu Q., Yan X., Li R., Yuan Y., Wang J., Zhao Y., Fu J., Su J. (2024). Polyamine Signal through Hcc Microenvironment: A Key Regulator of Mitochondrial Preservation and Turnover in Tams. Int. J. Mol. Sci..

[45] Pan J., Lin Z., Pan Q., Zhu T. (2025). Heterogeneity in Polyamine Metabolism Dictates Prognosis and Immune Checkpoint Blockade Response in Hepatocellular Carcinoma. Front. Immunol..

[46] Sun Y., Zhou P., Qian J., Zeng Q., Wei G., Li Y., Liu Y., Lai Y., Zhan Y., Wu D. (2025). Spermine Synthase Engages in Macrophages M2 Polarization to Sabotage Antitumor Immunity in Hepatocellular Carcinoma. Cell Death Differ..

[47] Zhou C., Basnet R., Zhen C., Ma S., Guo X., Wang Z., Yuan Y. (2024). Trimethylamine N-Oxide Promotes the Proliferation and Migration of Hepatocellular Carcinoma Cell through the Mapk Pathway. Discov. Oncol..

[48] Zhou Y., Zhang Y., Jin S., Lv J., Li M., Feng N. (2024). The Gut Microbiota Derived Metabolite Trimethylamine N-Oxide: Its Important Role in Cancer and Other Diseases. Biomed. Pharmacother..

[49] Wu Y., Rong X., Pan M., Wang T., Yang H., Chen X., Xiao Z., Zhao C. (2022). Integrated Analysis Reveals the Gut Microbial Metabolite Tmao Promotes Inflammatory Hepatocellular Carcinoma by Upregulating Postn. Front. Cell Dev. Biol..

[50] Zhang J., Sun Y., Yu M., Hu Y., Huang X., Yang G., Zhang R., Ge M. (2025). Tgf-Β/Smad Pathway Mediates Cadmium Poisoning-Induced Chicken Liver Fibrosis and Epithelial-Mesenchymal Transition. Biol. Trace Elem. Res..

[51] Wang X., Fang Y., Liang W., Cai Y., Wong C.C., Wang J., Wang N., Lau H.C., Jiao Y., Zhou X. (2025). Gut-Liver Translocation of Pathogen Klebsiella Pneumoniae Promotes Hepatocellular Carcinoma in Mice. Nat. Microbiol..

[52] Liu H., Qin C.Y., Han G.Q., Xu H.W., Meng M., Yang Z. (2007). Mechanism of Apoptotic Effects Induced Selectively by Ursodeoxycholic Acid on Human Hepatoma Cell Lines. World J. Gastroenterol..

[53] Zhu L., Shan L.J., Liu Y.J., Chen D., Xiao X.G., Li Y. (2014). Ursodeoxycholic Acid Induces Apoptosis of Hepatocellular Carcinoma Cells in Vitro. J. Dig. Dis..

[54] Chen Y., Zhang M., Liu Z., Zhang N., Wang Q. (2024). Ursodeoxycholic Acid Platinum(Iv) Conjugates as Antiproliferative and Antimetastatic Agents: Remodel the Tumor Microenvironment through Suppressing Jak2/Stat3 Signaling. J. Med. Chem..

[55] Chung G.E., Yoon J.H., Lee J.H., Kim H.Y., Myung S.J., Yu S.J., Lee S.H., Lee S.M., Kim Y.J., Lee H.S. (2011). Ursodeoxycholic Acid-Induced Inhibition of Dlc1 Protein Degradation Leads to Suppression of Hepatocellular Carcinoma Cell Growth. Oncol. Rep..

[56] Lee S., Cho Y.Y., Cho E.J., Yu S.J., Lee J.H., Yoon J.H., Kim Y.J. (2018). Synergistic Effect of Ursodeoxycholic Acid on the Antitumor Activity of Sorafenib in Hepatocellular Carcinoma Cells Via Modulation of Stat3 and Erk. Int. J. Mol. Med..

[57] Lim S.C., Choi J.E., Kang H.S., Han S.I. (2010). Ursodeoxycholic Acid Switches Oxaliplatin-Induced Necrosis to Apoptosis by Inhibiting Reactive Oxygen Species Production and Activating P53-Caspase 8 Pathway in Hepg2 Hepatocellular Carcinoma. Int. J. Cancer.

[58] Zhang L., Zheng Z., Huang H., Fu Y., Chen T., Liu C., Yi Q., Lin C., Zeng Y., Ou Q. (2024). Multi-Omics Reveals Deoxycholic Acid Modulates Bile Acid Metabolism Via the Gut Microbiota to Antagonize Carbon Tetrachloride-Induced Chronic Liver Injury. Gut Microbes.

[59] Xu Z., Huang G., Gong W., Zhou P., Zhao Y., Zhang Y., Zeng Y., Gao M., Pan Z., He F. (2012). Fxr Ligands Protect against Hepatocellular Inflammation Via Socs3 Induction. Cell Signal.

[60] Fuchs C.D., Trauner M. (2022). Role of Bile Acids and Their Receptors in Gastrointestinal and Hepatic Pathophysiology. Nat. Rev. Gastroenterol. Hepatol..

[61] Hu C., Xu B., Wang X., Wan W.H., Lu J., Kong D., Jin Y., You W., Sun H., Mu X. (2023). Gut Microbiota-Derived Short-Chain Fatty Acids Regulate Group 3 Innate Lymphoid Cells in Hcc. Hepatology.

[62] Li N., Niu L., Liu Y., Wang Y., Su X., Xu C., Sun Z., Guo H., Gong J., Shen S. (2024). Taking Scfas Produced by Lactobacillus Reuteri Orally Reshapes Gut Microbiota and Elicits Antitumor Responses. J. Nanobiotechnol..

[63] McBrearty N., Arzumanyan A., Bichenkov E., Merali S., Merali C., Feitelson M. (2021). Short Chain Fatty Acids Delay the Development of Hepatocellular Carcinoma in Hbx Transgenic Mice. Neoplasia.

[64] Jing G., Xu W., Ma W., Yu Q., Zhu H., Liu C., Cheng Y., Guo Y., Qian H. (2024). Echinacea Purpurea Polysaccharide Intervene in Hepatocellular Carcinoma Via Modulation of Gut Microbiota to Inhibit Tlr4/Nf-Κb Pathway. Int. J. Biol. Macromol..

[65] Jiang W., Zhu H., Xu W., Liu C., Hu B., Guo Y., Cheng Y., Qian H. (2021). Echinacea Purpurea Polysaccharide Prepared by Fractional Precipitation Prevents Alcoholic Liver Injury in Mice by Protecting the Intestinal Barrier and Regulating Liver-Related Pathways. Int. J. Biol. Macromol..

[66] Qin J., Huang X., Gou S., Zhang S., Gou Y., Zhang Q., Chen H., Sun L., Chen M., Liu D. (2024). Ketogenic Diet Reshapes Cancer Metabolism through Lysine Β-Hydroxybutyrylation. Nat. Metab..

[67] Ma X., FTian, Li J., Wu Z., Cao L. (2024). In Vitro Simulated Ketogenic Diet Inhibits the Proliferation and Migration of Liver Cancer Cells by Reducing Insulin Production and Down-Regulating Foxc2 Expression. Turk. J. Gastroenterol..

[68] Lan Y., Jin C., Kumar P., Yu X., Lenahan C., Sheng J. (2022). Ketogenic Diets and Hepatocellular Carcinoma. Front. Oncol..

[69] Cui X., Yun X., Sun M., Li R., Lyu X., Lao Y., Qin X., Yu W. (2023). Hmgcl-Induced Β-Hydroxybutyrate Production Attenuates Hepatocellular Carcinoma Via Dpp4-Mediated Ferroptosis Susceptibility. Hepatol. Int..

[70] Zhao L.Y., Li S.Y., Zhou Z.Y., Han X.Y., Li K., Xue S.T., Jiang J.D. (2024). Substituted Indole Derivatives as Unc-51-Like Kinase 1 Inhibitors: Design, Synthesis and Anti-Hepatocellular Carcinoma Activity. Biomed. Pharmacother..

[71] Qiao R., Fan X., Zhou L., Dong D., Liu Y., Liu D., Ma G., Tang N., Wang Y., Li X.Q. (2023). The Synthesis and Effects of a Novel Trpc6 Inhibitor, Bp3112, on Hepatocellular Carcinoma. Future Med. Chem..

[72] Lee C.M., Park S.H., Nam M.J. (2019). Anticarcinogenic Effect of Indole-3-Carbinol (I3c) on Human Hepatocellular Carcinoma Snu449 Cells. Hum. Exp. Toxicol..

[73] Lee C.M., Choi Y.J., Park S.H., Nam M.J. (2018). Indole-3-Carbinol Induces Apoptosis in Human Hepatocellular Carcinoma Huh-7 Cells. Food Chem. Toxicol..

[74] Wang X., He H., Lu Y., Ren W., Teng K.Y., Chiang C.L., Yang Z., Yu B., Hsu S., Jacob S.T. (2015). Indole-3-Carbinol Inhibits Tumorigenicity of Hepatocellular Carcinoma Cells Via Suppression of Microrna-21 and Upregulation of Phosphatase and Tensin Homolog. Biochim. Biophys. Acta.

[75] Abdelmageed M.M., El-Naga R.N., El-Demerdash E., Elmazar M.M. (2016). Indole-3- Carbinol Enhances Sorafenib Cytotoxicity in Hepatocellular Carcinoma Cells: A Mechanistic Study. Sci. Rep..

[76] Yu C., Liu R., Xie C., Zhang Q., Yin Y., Bi K., Li Q. (2015). Quantification of Free Polyamines and Their Metabolites in Biofluids and Liver Tissue by Uhplc-Ms/Ms: Application to Identify the Potential Biomarkers of Hepatocellular Carcinoma. Anal. Bioanal. Chem..

[77] Yue F., Li W., Zou J., Jiang X., Xu G., Huang H., Liu L. (2017). Spermidine Prolongs Lifespan and Prevents Liver Fibrosis and Hepatocellular Carcinoma by Activating Map1s-Mediated Autophagy. Cancer Res..

[78] Goyal L., Supko J.G., Berlin J., Blaszkowsky L.S., Carpenter A., Heuman D.M., Hilderbrand S.L., Stuart K.E., Cotler S., Senzer N.N. (2013). Phase 1 Study of N(1),N(11)-Diethylnorspermine (Denspm) in Patients with Advanced Hepatocellular Carcinoma. Cancer Chemother. Pharmacol..

[79] Enjoji M., Nakamuta M., Arimura E., Morizono S., Kuniyoshi M., Fukushima M., Kotoh K., Nawata H. (2004). Clinical Significance of Urinary N1,N12-Diacetylspermine Levels in Patients with Hepatocellular Carcinoma. Int. J. Biol. Markers.

[80] Kawakita M., Hiramatsu K., Sugimoto M., Takahashi K., Toi M. (2004). Clinical Usefulness of Urinary Diacetylpolyamines as Novel Tumor Markers. Rinsho Byori.

[81] Al-Malki A.L., Razvi S.S., Mohammed F.A., Zamzami M.A., Choudhry H., Kumosani T.A., Balamash K.S., Alshubaily F.A., ALGhamdi S.A., Abualnaja K.O. (2019). Synthesis and in Vitro Antitumor Activity of Novel Acylspermidine Derivative N-(4-Aminobutyl)-N-(3-Aminopropyl)-8-Hydroxy-Dodecanamide (Aahd) against Hepg2 Cells. Bioorg Chem..

[82] Wang Y., Qiu Z., Zhou B., Liu C., Ruan J., Yan Q., Liao J., Zhu F. (2015). In Vitro Antiproliferative and Antioxidant Effects of Urolithin a, the Colonic Metabolite of Ellagic Acid, on Hepatocellular Carcinomas Hepg2 Cells. Toxicol In Vitro.

[83] Djedjibegovic J., Marjanovic A., Panieri E., Saso L. (2020). Ellagic Acid-Derived Urolithins as Modulators of Oxidative Stress. Oxid. Med. Cell Longev..

[84] Zhang H., Li C., Han L., Xiao Y., Bian J., Liu C., Gong L., Liu Z., Wang M. (2024). Mup1 Mediates Urolithin a Alleviation of Chronic Alcohol-Related Liver Disease Via Gut-Microbiota-Liver Axis. Gut Microbes.

[85] Zhang H., Zhou W., Gao P., Li Z., Li C., Li J., Bian J., Gong L., He C., Han L. (2024). Ellagic Acid Protects against Alcohol-Related Liver Disease by Modulating the Hepatic Circadian Rhythm Signaling through the Gut Microbiota-Npas2 Axis. J. Agric. Food Chem..

[86] Lv M.Y., Shi C.J., Pan F.F., Shao J., Feng L., Chen G., Ou C., Zhang J.F., Fu W.M. (2019). Urolithin B Suppresses Tumor Growth in Hepatocellular Carcinoma through Inducing the Inactivation of Wnt/Β-Catenin Signaling. J. Cell Biochem..

[87] Song Q., Zhang X., Liu W., Wei H., Liang W., Zhou Y., Ding Y., Ji F., Cheung A.H.-K., Wong N. (2023). Bifidobacterium Pseudolongum-Generated Acetate Suppresses Non-Alcoholic Fatty Liver Disease-Associated Hepatocellular Carcinoma. J. Hepatol..

[88] Aoki R., Onuki M., Hattori K., Ito M., Yamada T., Kamikado K., Kim Y.G., Nakamoto N., Kimura I., Clarke J.M. (2021). Commensal Microbe-Derived Acetate Suppresses Nafld/Nash Development Via Hepatic Ffar2 Signalling in Mice. Microbiome.

[89] Lau H.C., Zhang X., Ji F., Lin Y., Liang W., Li Q., Chen D., Fong W., Kang X., Liu W. (2024). Lactobacillus Acidophilus Suppresses Non-Alcoholic Fatty Liver Disease-Associated Hepatocellular Carcinoma through Producing Valeric Acid. EBioMedicine.

[90] Yu J., Zhu P., Shi L., Gao N., Li Y., Shu C., Xu Y., Yu Y., He J., Guo D. (2024). Bifidobacterium Longum Promotes Postoperative Liver Function Recovery in Patients with Hepatocellular Carcinoma. Cell Host Microbe.

[91] Zhang X., Coker O.O., Chu E.S., Fu K., Lau H.C.H., Wang Y.X., Chan A.W.H., Wei H., Yang X., Sung J.J.Y. (2021). Dietary Cholesterol Drives Fatty Liver-Associated Liver Cancer by Modulating Gut Microbiota and Metabolites. Gut.

[92] Chen S., Han P., Zhang Q., Liu P., Liu J., Zhao L., Guo L., Li J. (2023). Lactobacillus Brevis Alleviates the Progress of Hepatocellular Carcinoma and Type 2 Diabetes in Mice Model Via Interplay of Gut Microflora, Bile Acid and Notch 1 Signaling. Front. Immunol..

[93] Wang J.W., Kuo C.H., Kuo F.C., Wang Y.K., Hsu W.H., Yu F.J., Hu H.M., Hsu P.I., Wang J.Y., Wu D.C. (2019). Fecal Microbiota Transplantation: Review and Update. J. Formos. Med. Assoc..

[94] Routy B., Le Chatelier E., Derosa L., Duong C.P.M., Alou M.T., Daillère R., Fluckiger A., Messaoudene M., Rauber C., Roberti M.P. (2018). Gut Microbiome Influences Efficacy of Pd-1-Based Immunotherapy against Epithelial Tumors. Science.

[95] Matson V., Fessler J., Bao R., Chongsuwat T., Zha Y., Alegre M.L., Luke J.J., Gajewski T.F. (2018). The Commensal Microbiome Is Associated with Anti-Pd-1 Efficacy in Metastatic Melanoma Patients. Science.

[96] Gopalakrishnan V., Spencer C.N., Nezi L., Reuben A., Andrews M.C., Karpinets T.V., Prieto P.A., Vicente D., Hoffman K., Wei S.C. (2018). Gut Microbiome Modulates Response to Anti-Pd-1 Immunotherapy in Melanoma Patients. Science.

[97] Ye X., Zhang T., Han H. (2022). Pparα: A Potential Therapeutic Target of Cholestasis. Front. Pharmacol..

[98] Zhang X., Shi L., Lu X., Zheng W., Shi J., Yu S., Feng H., Yu Z. (2024). Bile Acids and Liver Cancer: Molecular Mechanism and Therapeutic Prospects. Pharmaceuticals.

[99] Chen Y., Wang Y., Lei J., Chen B., Zhang X., Chang L., Hu Z., Wang Y., Lu Y. (2024). Taurohyocholic Acid Acts as a Potential Predictor of the Efficacy of Tyrosine Kinase Inhibitors Combined with Programmed Cell Death-1 Inhibitors in Hepatocellular Carcinoma. Front. Pharmacol..

[100] He L., Li Z., Su D., Du H., Zhang K., Zhang W., Wang S., Xie F., Qiu Y., Ma S. (2024). Tumor Microenvironment-Responsive Nanocapsule Delivery Crispr/Cas9 to Reprogram the Immunosuppressive Microenvironment in Hepatoma Carcinoma. Adv. Sci..

[101] Coutzac C., Jouniaux J.M., Paci A., Schmidt J., Mallardo D., Seck A., Asvatourian V., Cassard L., Saulnier P., Lacroix L. (2020). Systemic Short Chain Fatty Acids Limit Antitumor Effect of Ctla-4 Blockade in Hosts with Cancer. Nat. Commun..

[102] Facciorusso A., Del Prete V., Crucinio N., Muscatiello N., Carr B.I., Di Leo A., Barone M. (2015). Angiotensin Receptor Blockers Improve Survival Outcomes after Radiofrequency Ablation in Hepatocarcinoma Patients. J. Gastroenterol. Hepatol..

[103] Ballester M.P., Abril C., Merino V., Alós M., Segarra G., Tosca J., Montón C., Casasus N., Lluch P. (2024). Atezolizumab Plus Bevacizumab Treatment for Unresectable Hepatocellular Carcinoma: Real-Life Experience from a Single Tertiary Centre in Spain and Albi Score as a Survival Prognostic Factor. Cancer Diagn. Progn..

[104] Hatanaka T., Kakizaki S., Hiraoka A., Tada T., Hirooka M., Kariyama K., Tani J., Atsukawa M., Takaguchi K., Itobayashi E. (2024). Comparative Analysis of the Therapeutic Outcomes of Atezolizumab Plus Bevacizumab and Lenvatinib for Hepatocellular Carcinoma Patients Aged 80 years and Older: Multicenter Study. Hepatol. Res..

[105] Zhou A., Tang L., Zeng S., Lei Y., Yang S., Tang B. (2020). Gut Microbiota: A New Piece in Understanding Hepatocarcinogenesis. Cancer Lett..

[106] Iida N., Dzutsev A., Stewart C.A., Smith L., Bouladoux N., Weingarten R.A., Molina D.A., Salcedo R., Back T., Cramer S. (2013). Commensal Bacteria Control Cancer Response to Therapy by Modulating the Tumor Microenvironment. Science.

[107] Pallozzi M., De Gaetano V., Di Tommaso N., Cerrito L., Santopaolo F., Stella L., Gasbarrini A., Ponziani F.R. (2024). Role of Gut Microbial Metabolites in the Pathogenesis of Primary Liver Cancers. Nutrients.

[108] Zheng Y., Wang T., Tu X., Huang Y., Zhang H., Tan D., Jiang W., Cai S., Zhao P., Song R. (2019). Gut Microbiome Affects the Response to Anti-Pd-1 Immunotherapy in Patients with Hepatocellular Carcinoma. J. Immunother. Cancer.

[109] Oh B., Boyle F., Pavlakis N., Clarke S., Eade T., Hruby G., Lamoury G., Carroll S., Morgia M., Kneebone A. (2021). The Gut Microbiome and Cancer Immunotherapy: Can We Use the Gut Microbiome as a Predictive Biomarker for Clinical Response in Cancer Immunotherapy?. Cancers.

[110] Shah D.D., Chorawala M.R., Raghani N.R., Patel R., Fareed M., Kashid V.A., Prajapati B.G. (2025). Tumor Microenvironment: Recent Advances in Understanding and Its Role in Modulating Cancer Therapies. Med. Oncol..

[111] Wu X.Q., Ying F., Chung K.P.S., Leung C.O.N., Leung R.W.H., So K.K.H., Lei M.M.L., Chau W.K., Tong M., Yu J. (2025). Intestinal Akkermansia Muciniphila Complements the Efficacy of Pd1 Therapy in Mafld-Related Hepatocellular Carcinoma. Cell Rep. Med..

[112] Qu D., Chen M., Zhu H., Liu X., Cui Y., Zhou W., Zhang M. (2023). Akkermansia Muciniphila and Its Outer Membrane Protein Amuc_1100 Prevent High-Fat Diet-Induced Nonalcoholic Fatty Liver Disease in Mice. Biochem. Biophys. Res. Commun..

[113] Sparfel L., Ratodiarivony S., Boutet-Robinet E., Ellero-Simatos S., Jolivet-Gougeon A. (2024). Akkermansia Muciniphila and Alcohol-Related Liver Diseases. A Systematic Review. Mol. Nutr. Food Res..

